# A New Algorithm for Estimating a Noiseless, Evenly Sampled, Heart Rate Modulating Signal

**DOI:** 10.3390/bioengineering10050552

**Published:** 2023-05-04

**Authors:** Enrico M. Staderini, Harish Kambampati, Amith K. Ramakrishnaiah, Stefano Mugnaini, Andrea Magrini, Sandro Gentili

**Affiliations:** 1 Healthy World Association Switzerland, 1400 Yverdon-les-Bains, Switzerland; 2 Doctorate School in Industrial Engineering, “Tor Vergata” University of Rome, 00133 Roma, Italy; 3 Department of Electronics Engineering, “Tor Vergata” University of Rome, 00133 Roma, Italy; 4 Occupational Medicine Section, Department of Biomedicine and Prevention, “Tor Vergata” University of Rome, 00133 Roma, Italy

**Keywords:** heart rate variability, signal analysis

## Abstract

Heart rate variability (HRV) is commonly intended as the variation in the heart rate (HR), and it is evaluated in the time and frequency domains with various well-known methods. In the present paper, the heart rate is considered as a time domain signal, at first as an abstract model in which the HR is the instantaneous frequency of an otherwise periodic signal, such as with an electrocardiogram (ECG). In this model, the ECG is assumed to be a frequency modulated signal, or carrier signal, where HRV or HRV(t) is the time-domain signal which is frequency modulating the carrier ECG signal around its average frequency. Hence, an algorithm able to frequency demodulate the ECG signal to extract the signal HRV(t) is described, with possibly enough time resolution to analyse fast time-domain variations in the instantaneous HR. After exhaustive testing of the method on simulated frequency modulated sinusoidal signals, the new procedure is eventually applied on actual ECG tracings for preliminary nonclinical testing. The purpose of the work is to use this algorithm as a tool and a more reliable method for the assessment of heart rate before any further clinical or physiological analysis.

## 1. Introduction

In this paper, the authors assume that the variation of the heart rate, hence the frequency variation of the ECG, is a time domain signal per se, which frequently modulates the ECG. This model assumption is similar to frequency modulated (FM) radio broadcasting where an audio signal is used to frequency modulate the radio frequency carrier. Frequency demodulation for recovering the audio signal, at the radio receiver end, is a straightforward and well-known process, but it appears to be very tricky and put to its physical limits, when considered for the heart rate “carrier” and, in our case, when a large bandwidth of the recovered modulation signal is needed to be preserved.

**Figure 1:**
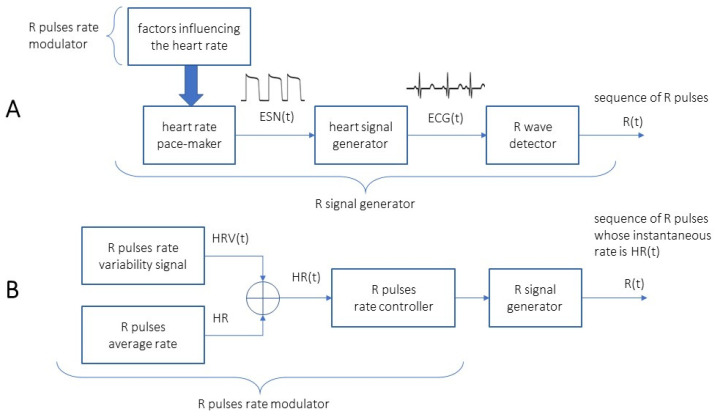
An abstract and almost black box model of the heart rate variability signal as a heart rate frequency modulator. (**A**) A black box model of any physiologic factor (R pulse rate modulator shown upper left) is blindly considered to impose a certain discharge frequency on the cells in the sinus node, which provide an electrical sinus node signal ESN(t) that propagates in the heart, and eventually will be responsible for producing an electrical signal ECG(t) recorded on the skin of the body. An external R-wave detector produces a signal R(t) that is zero at any instant of time, but when an R-wave is detected so its value is set to one (the detected R pulse). (**B**): The R pulse rate modulator is now black box modelled as an R pulse rate variability signal generator that produces a signal that is equal to the variation of the heart rate (over the average) at each instant of time HRV(t). This signal is then summed to a constant value HR representing the average heart rate in the considered epoch to produce a signal that is equal to the heart rate at each instant of time HR(t). All of the time-domain signals indicated in the figure are assumed to be continuous in time.

Discussion of the origin of heart rate modulations or heart rate variability is outside the scope of this paper. The new method completely disregards the physiology of the heart and the mechanisms that influence and define the heart rate in real time. It is not the intention of the authors to investigate the physiology of the heart or to propose any new insight into it; on the contrary, a method is proposed for extracting a clean heart rate variability signal from a heart-activity-related signal such as an electrocardiogram (ECG). Our interest in the physiology of the phenomena implied in heart rate control is limited to the understanding that the ECG is a frequency modulated signal (the times of occurrence of R-waves are not evenly spaced in time), thus one is looking for a continuous time signal reflecting the inverse of the instantaneous time delay between the R-wave events, and this signal is called the instantaneous heart rate, and indicated as HR(t). This signal is not to found solely in any particular organ or physiologic mechanism in the body; in this paper it is considered only from a black box point of view, as depicted in Figure 1.

Contrary to commonly known HRV analysis methods, in this paper the authors are looking for an algorithm that is able to detect very fast variations in the heart rate or fast, non-rhythmic variations intervening in a few heart cycles. In a nutshell, our method is going to demodulate the signal frequency R(t) to recover the assumed frequency modulating signal HRV(t). The latter signal, no matter if it is periodic or not, will then be used for the study of any particular heart rate variability analysis that might be required.

The aim and need of the new algorithm presented here will be better understood in comparing its results with those obtained with more common interbeat interval plotting followed by interpolation/smoothing. 

### 1.1. Intrinsic Signal Analysis Problems in the Frequency Demodulation of the ECG Signal

Comparing the frequency band occupation of a radio FM signal with the frequency occupation of the ECG(t) signal (simplifying the ECG as a sinusoidal signal to also avoid the spectral content of its waveform) may be very instructive in highlighting the originality and importance of this new method.

It is important to go through the basics of FM radio to understand the difficulty in the recovering of HRV(t) from the ECG(t) signal.

FM radio broadcasting is very popular (although it has now become obsolete with the introduction of digital audio broadcasting, or DAB). In the famous 88–108 MHz radio band, many FM stations are allocated with a spacing of 200 kHz, while a maximum modulation deviation of ±75 kHz is permitted for sending stereo audio signals in a band from tens of Hz to 50 kHz (also considering stereo multiplexing). It is very well-known that the intrinsic non-linearity of the frequency modulation process makes the calculation of the occupied band of the modulated signal very difficult to assess. The popular Carson bandwidth rule [1] gives a rough estimation of the bandwidth (Carson Bandwidth or CB) of a signal frequency modulated by another signal having a maximum frequency $f_{m}$ with a maximum permitted frequency deviation of the carrier Δf from the non-modulated carrier frequency:

(1) $$CB=2\cdot \left(f_{m}+\Delta f\right)$$

For an FM radio emission with $f_{m}$ = 53 kHz and Δf = 75 kHz, one gets CB = 256 kHz, which is even a bit larger than the allocated band for each FM radio channel.It is worthy of note that the occupied band is just 0.26 percent of the carrier (at the center band of 98 MHz).

The same calculation for the frequency modulated ECG signal is quite astonishing: first, one may assume that the possible absolute maximum deviation of the heart rate from a given average (normal) heart rate would be in the order of 40 beats per minute (bpm) or 0.7 Hz, meaning that one might expect, for example, for the heart rate running at 70 bpm (1.17 Hz) to slow down to 30 bpm (0.5 Hz) or rise to 110 bpm (1.8 Hz) although this is just an assumption that uses approximate figures; second, the maximum frequency of the variation might be assumed to be a bit larger than breath frequency, which is a well-known modulator of the heart rate, for instance a maximum of 15 events per minute (0.25 Hz). This is just for the sake of having some acceptable figures to let us compute the CB. Thus, for the heart rate of 70 bpm (1.17 Hz), one has $f_{m}$ = 0.25 Hz and Δf = 0.7 Hz, which gives CB = 1.9 Hz, explaining the intrinsic difficulty or even impossibility of a correct estimation of the HRV, as the signal modulating the heart rate has a larger bandwidth than the carrier (average heart rate) itself. Indeed, in the calculation above, they were considered as maximum values, as it is uncommon for the breath-frequency-induced HRV to have a shift of 40 bpm. Furthermore, the problem remains, as the CB of the heart rate signal is still comparable to the average heart rate itself.

### 1.2. Competing Methods for the Frequency Demodulation of the ECG Signal

In conclusion, it must be admitted that the heart is running too slowly to correctly carry the expected heart rate variability modulation; hence, it can be said that, in general, the detection of the heart rate variability signal from any heart-activity-related physiologic signal, such as the ECG, is conceptually impossible. It may be surmised that this problem is the basic reason for the large number of methods [2], and their differences, that have been utilized so far for heart rate variability assessment and analysis.

The concepts previously highlighted make the use of standard frequency demodulators, commonly used in radio technology, impossible. Indeed, HRV has always been evaluated by computing the actual time delays of the arrival of each individual heartbeat or instantaneous heart period, starting from the interbeat interval values.

There are two problems to underline. First, no matter the demodulation process used to estimate HRV(t), a continuous heart rate modulation signal will never be obtained, as the instantaneous heart rate is not available at each instant of time but only at each heartbeat (by computing the inverse of the instantaneous heart period). Thus, the heart rate variability signal, which one assumes in the black box model as a signal that is continuous in nature, is not available in a continuous form (as with the blood pressure signal, for example). Second, it is true that biological signals are always numerically acquired in a sampled form, but the sampling frequency is normally chosen by the experimenter, while with the HRV(t) signal, one should follow the sampling frequency of the signal (which is the heart period) and the heart period, or heart rate, is exactly what the experimenter is looking for. So, the visible HRV signal is in general a non-continuous signal available at its own sampling frequency, which is, by the way, the signal itself.

It is the opinion of the authors that there is no need to review all of the HRV acquisition and analysis techniques that have been developed so far. All of these algorithms can be roughly divided into long-term and short-term HRV analysis methods depending on the length of the ECG epoch to be studied. The results can also be divided into time- or frequency-based analyses. The readers well acquainted in this field know the story very well. For the other interested readers, reference [2] is suggested.

As explained above, the method described in this paper is now in need of a reliable HRV(t) signal, and not for its physiological meaning. Nevertheless, it is important to discuss and review the current state of research in the field, with special attention devoted to the papers dealing with the problem of HRV signal detection, recording, and resampling. Of special interest for our work is the paper by [3,4,5], where the effects of resampling the frequency of the RR interval and the length of the epoch of analysis are considered with regard to the evaluation of the autonomic nervous system. Rapid variations of the acceleration and deceleration of the heart rate were also analyzed in [6,7,8], where interest was given to the preprocessing of unevenly sampled RR interval signals using interpolation and resampling.

With regard to comparing HRV analysis methods, it is important to mention the Lomb-Scargle periodogram [7,8,9,10,11], which can be used for a reliable estimation of the frequency spectrum of unevenly sampled signals such as the RR series. The Lomb-Scargle periodogram is more common within the astronomical research community, but it is, quite unfortunately, less popular in the HRV research community, mostly because of the mathematical difficulties in understanding it. In any case, Lomb-Scargle is looking for rhythmical variations in the RR series, while it is better to remain general and not assume any special behavior of the HR modulating signal. This means that at this moment one is not looking at the frequency of variation of the heart rate, as no assumption of stationarity can be used on it.

The problem of resampling and the actual final sampling rate of the HRV signal has also been studied in [12,13,14], where another time domain measure of HRV has been introduced. Errors and effects introduced in the resampling process of HRV were also studied in other papers [15,16,17,18,19,20].

The present paper introduces yet another method for obtaining a resampled and clean HRV signal out of a sequence of RR intervals, with the aim of preserving the bandwidth of the frequency-modulating signal, even on short or ultra-short time epochs.

## 2. Materials and Methods

### 2.1. Algorithm for the Estimation of the Instantaneous Heart Rate Signal from the ECG

As already stated, the presented algorithm aims to obtain a signal representative of the “instantaneous” heart rate. This signal should be provided with a sampling rate even higher than the heart rate itself.

There are two standard and straightforward methods of extracting the heart rate signal from the ECG signal [2]; both methods start with measuring each heart period and convert it to frequency by calculating the inverse. The resulting collection of measures will normally be unevenly spread in time as the time delay between a measure and the following is only equal to each heart period, which varies. Thus, one may say that the HR(t) signal is available only in an unevenly sampled fashion, where the actual sampling is the signal itself. To create an evenly sampled, resampled heart rate signal extracted from the set of measures, the first way is by distributing each sample measure on a reconstructed sampling frequency equal to the average heart rate in the epoch to be analyzed. The second method involves leaving the measures at the instants of time where they were taken, and interpolating them on an evenly distributed instant of time. The same interpolation is to be done when using the first method if a larger number of points is required. This problem is commonly known as resampling. Both methods will provide an HR(t) signal suitable for subsequent analysis in the frequency domain, but is relatively poor for direct study in the time domain. Indeed, time-domain studies of HR(t) are almost never done on the waveform of HR(t) but, more often, are performed on indexes extracted from it [2].

The method used in this paper aims to obtain a signal HR(t) that might be directly analyzed for the study of fast transitory events appearing in the signal itself. Indeed, the final goal and the advantage of the algorithm about to be described is that it can catch rapid variations in the heart rate, although they are not rhythmical, which might be related, for example, to sudden correlated variations in the activity of the autonomic nervous system.

Thus, the problem to be tackled is that of estimating the signal HRV(t), or HR(t), from the ECG(t). At first, it must recognized that neither HRV(t) nor HR(t) can be directly measured with appreciable time resolution. Indeed, even assuming that one had a reliable R-wave detector on the signal ECG(t), one could just measure HR(t) at each nth heartbeat occurring at the instant of time $t_{n}$. It is worthy of note that the HR(t) signal is the only biological signal that is naturally available in sampled form; moreover, as already noted above, its sampling instants of time are the signal itself.

In this new proposed method, a new signal SR(t) is first built as the cumulative sum of the heart beats (or complete ECG(t) cycles). A suitable, evenly spaced sampling time $t_{s}$ for the SR(t) signal is defined. This means that the SR(t) will be created as a signal sampled at a frequency of our choice: $f_{s}=\frac{1}{t_{s}}$. A convenient sampling frequency for $f_{s}$ is chosen which is often even higher than the actual heart rate, and certainly much higher than the frequency content of HR(t), and perhaps one will even use the same sampling frequency at which the signal ECG(t) itself was acquired. Next, at each instant of sampling, the following rule is applied:

If a heartbeat (R-wave) has occurred (has been detected) at an instant of time $t_{n}$, where $t_{i-1}<t_{n}⩽t_{i}$, then $SR\left(t_{i}\right)=SR\left(t_{i-1}\right)+1$, otherwise $SR\left(t_{i}\right)=SR\left(t_{i-1}\right)$.

This means that SR(t) will be built as the running count function of the detected heartbeat. It is obvious that it will be a not-descending function of time; it will be sampled at the frequency $f_{s}$ with an obvious [instantaneous] slope equal to HR(t) beats per minute. Indeed, the word “instantaneous” is used in brackets because the signal SR(t) is a sampled staircase signal which remains constant during many time samples between each heartbeat and that following it. Note that the signal SR(t) rises as faster as higher is the actual signal HR(t): indeed, supposing HR(t) be constant at 70 bpm, then SR(t) will obviously grow at a rate of 70 units per minute.

Thus, HR(t) may be evaluated as just the first derivative of SR(t):

(2) $$HR\left(t\right)=\frac{dSR\left(t\right)}{dt}\;\mathrm{in}\;\mathrm{bpm}.$$

As $HR\left(t\right)=HRV\left(t\right)+\bar{f_{0}}$, the signal’s average (mean heart rate) may be subtracted to obtain $HRV\left(t\right)=HR\left(t\right)-f_{0}$ in bpm.

Apart from a series of difficulties which will be discussed, it is worth noting that the estimated HRV(t) signal is in principle known with a remarkable time resolution (it is sampled at a very high sampling frequency), and hence it is helpful for ultra-short term heart rate variability (USTHRV) studies. Such studies are also useful in the detection of fast and short-lived heart rate variations (time domain HRV studies), provided that the required minimum signal epoch is available.

The authors are convinced that this proposed method is better than the standard method which is based on the interpolation of the inverse of individual heart periods.

The attentive reader will note that computing the signal SR(t) and then making the derivative of it is somewhat like performing the inverse of the interbeat interval values followed by interpolation or smoothing. In the present proposed method, the interpolation is still used on the signal SR(t) before computing the derivative, and it is also done through low pass filtering, as described below. Nevertheless, the obtained signal still maintains a wide frequency content that may show rapid heart rate variations, along with resampling at a high sampling frequency.

The details of the method will be presented here while applying the algorithm to a special artificial simulated signal which is used to stress and also to better appreciate the results and performances of the proposed processing.

The central assumption put forward in this paper is that any heart activity-related biomedical signal (electrocardiogram ECG, phonocardiogram PCG, photoplethysmogram PPG, etc.) is a frequency-modulated signal, while its instantaneous frequency is known as the instantaneous heart rate. Considering the ECG, whose instantaneous frequency is time-varying, the instantaneous frequency is a time signal. It is useful here to recall the definition of a “signal” as “any observable change of a quantity over time” [21]. That is why the heart rate signal has to be a signal indicated as HR(t). The objective of this paper is to describe a method for the frequency demodulation of the ECG to obtain an estimation of the instantaneous heart rate signal. The carrier signal has a sinusoidal shape in conventionally frequency-modulated signals, such as those used in radio communications. In the case at hand, the heart-activity-related biomedical signal is not sinusoidally shaped. Indeed, the ECG is not sinusoidal, although it may be considered as a periodic signal as well, made of repetitions of the well-known P-Q-R-S-T waves instead of sinusoidally shaped waves. Now, no matter the actual frequency content of the biomedical signal, which depends on its wave shape, the signal with its periodism is considered as the carrier of a frequency-modulating signal in the time domain, which might be named as the heart rate signal. The heart rate signal is not directly available for acquisition in the real world. However, it is known that the heart rate signal is visible as the instantaneous frequency of other signals generated synchronously with heart activity, the electrocardiogram being an example of this.

As the ECG has a large bandwidth relative to other heart-activity-related signals, and given that it is simple to process the determination of the heart rate by the detection of R-waves, the heart rate is commonly inferred from the frequency of the ECG events, as its jitter incertitude is at the minimum. Jitter may be defined as the deviation from the true periodicity of a presumably periodic signal [22]. As the ECG is, by its nature, not periodic, it is evident that the jitter, in assessing the true instant of time at which the R-wave in the ECG is detected, will be made of two components: one component is the natural (physiological) wandering of the heart rate (the scope of our research), and the second component is the instrumental error in detecting the time of arrival of the R-wave (the real instant of the heartbeat event). The first component is the result of the heart rate variability signal (also made of two components: the real wandering of the cardiac physiological pacemaker, which generates P-waves, summed to the wandering of the impulse transmission delay from the physiological pacemaker through the specific heart conduction tissues to the ventricles, which generates R-waves), and the second component is an unavoidable instrumental error. Indeed, an ECG is used thanks to its large bandwidth, and thus on this signal the instrumental error can be kept to a minimum compared to other heart-activity-related biological signals. At the end of the preliminary description, the ECG signal will be used to infer the shape of the heart rate signal, and the instrumental jitter will always be assumed to be zero.

**Figure 2:**
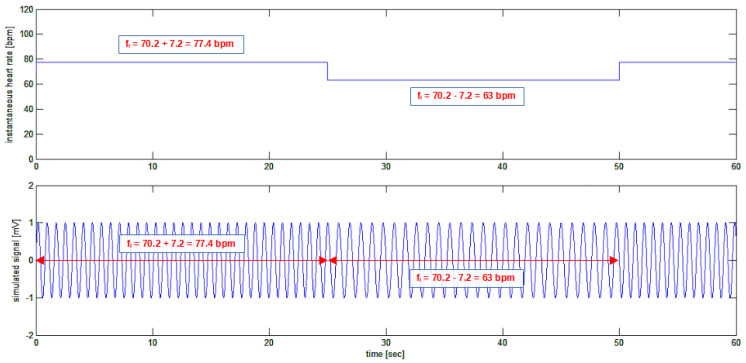
Upper tracing: the simulation of an artificial instantaneous “heart rate signal”, or HR(t), composed of a constant signal with an amplitude of 70.2 bpm summed to a square-wave signal having a peak-to-peak amplitude of 14.4 bpm and a frequency of 0.02 Hz; the resulting signal is a square-wave signal having an average value of 70.2 bpm alternating between 77.4 bpm and 63 bpm every 25 s. Lower trace: the HR(t) signal imposes the frequency of a sinusoidal signal, creating a “sinusoidal ECG” signal whose frequency is, at each instant of time, precisely that shown in the upper trace. Note that the amplitude of the HR(t) signal is purposely given in beats per minute (bpm), while the units of the simulated “sinusoidal ECG” signal are arbitrary. The frequency of the sinusoidal signal has been modulated by the square signal.

The ECG is obviously considered as the time-domain signal produced by the ventricles’ electrical activation: ECG(t). To better describe our model and without loss of generality, a sinusoidal signal is initially considered as a classical kind of periodic signal that is clearly simpler to manage than the ECG shape itself. Therefore, we will first consider a sinusoidal signal rather than an ECG signal. As one is interested in the wandering of the frequency of repetitions, the shape of what repeats itself is meaningless. To better understand the matter, one can start from Figure 2, where the artificially generated “heart rate signal” is shown.

It may come as a surprise that a square wave is used for simulating the heart rate signal in Figure 2, as heart rate variation never has this shape in nature. Indeed, the choice of a square wave, having a sudden and perpendicular rise in the waveform, has been made to show and test the performance of the algorithm in detecting fast transients in the heart rate variability signal, even if a so rapid a variation is very unlikely to happen in nature.

### 2.2. Practical Implementation of the Algorithm

A deeper insight is now given into the details of the practical implementation of the algorithm described above. The method is initially used on an artificial simulated signal showing a sudden variation in its frequency. The signal used is of sinusoidal shape because it is much simpler to simulate than an actual ECG-shaped signal. Therefore, the R detector will be temporarily changed in favor of a positive going zero-crossing detector.

Thus, the first step in FM demodulation of the ECG signal is detecting the signal cycles. In the case of the simulated ECG signal (sinusoidal shape), positive zero-crossings are detected, while in the case of a real ECG signal, the peaks of the R-wave will be detected. A positive zero-crossing event in the signal is detected at the instant of time when the signal is crossing the zero-line, traveling from a negative value to a positive value (positive first derivative). As the signal is artificially generated without any noise component, the following very simple MATLAB function has been written for zero-crossing detection:
function pf = zerofind(y) % zerofind function % % This function finds all the positive zero-crossings in the series % of values passed by y which is a row vector with values to explore for % zero-crossings % the function returns a two columns matrix %—first column contains the indexes where zeroes were found, %—second columns contains values of y vector at those indexes % size(pf,1) is the number of zeroes found % pf=[]; tdold=0; iold=1; for i=2:size(y,2)   if (y(i-1)<0) && (y(i)>0)     td=i-iold;     if (td-tdold)>0       pf=[pf; i-1 y(i)];       tdold=td;     end   end end

**Figure 3:**
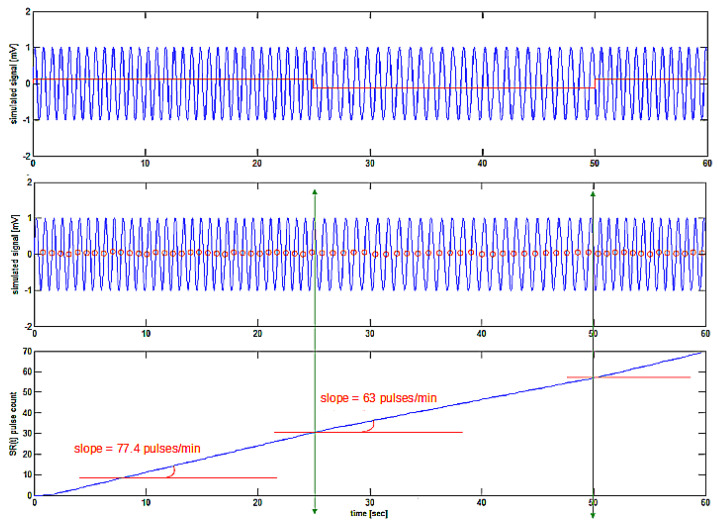
The upper trace shows a frequency-modulated signal with the square-wave modulating signal superimposed in red. Zero-crossing detection points (red) on the signal ECG(t) are shown in the middle trace. Bottom tracing shows the created SR(t) signal with a slope depending on the local instantaneous frequency of the simulated ECG(t) signal.

In the following, the simulated signal as described in Figure 2 is used. Using the zerofind(y) function, the zeroes in the signal are found, and, after this zero-crossing detection, the signal is created by the cumulative sum of the zero-crossing events in time, as shown in Figure 3.

The method for obtaining the cumulative sum signal implies counting the number of cycles detected over time using the following MATLAB script:



% events cumulative summing (or counting of complete cycles)



% vector peaks is the output of the zerofind function



%


sumecg=[0]; % initialize the cumulative sum count signal

j=1;     % initialize index on the peaks matrix

for i=2:(size(ecg,2)-1) % running over the ecg values

  if i>peaks(j,1) % present index just passed event position


    % event found, increment cumulative counter and add


    sumecg=[sumecg sumecg(size(sumecg,2))+1];

    if i<peaks(size(peaks,1),1) % go on if event not found

      j=j+1;


    else


      break; % exit for


    end



  else


    % no event found

    sumecg=[sumecg sumecg(size(sumecg,2))];


  end



end



In the above MATLAB script, the ecg vector is the simulated frequency-modulated signal (or the sampled ECG epoch), and the same vector is passed to the zerofind function above.

The sumecg vector obtained merits some discussion in order to be clearly understood. As already described, it is the summing count of the cycles (zero crossings in the simulated signal or R-waves detected in the ECG epoch). In Figure 3, it is shown as a rising straight line. However, it must be noted that the higher the number of cycles (or R-waves) detected per unit time, the steeper will be the slope of the sumecg vector (which, by the way, is the SR(t) signal), so it is not really a straight line. This means that the first derivative of the sumecg vector, or the SR(t) signal, should give the instantaneous signal frequency (or heart rate).

**Figure 4:**
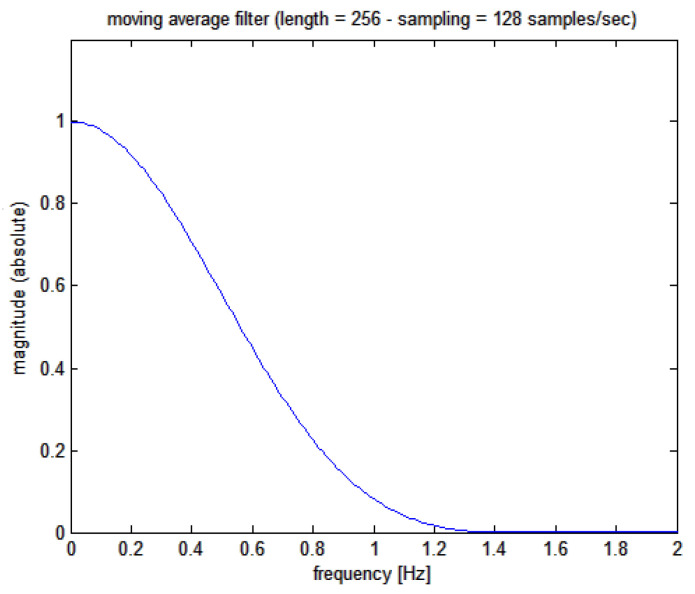
Magnitude Bode plot of the moving average filter applied to the cumulative sum signal.

**Figure 5:**
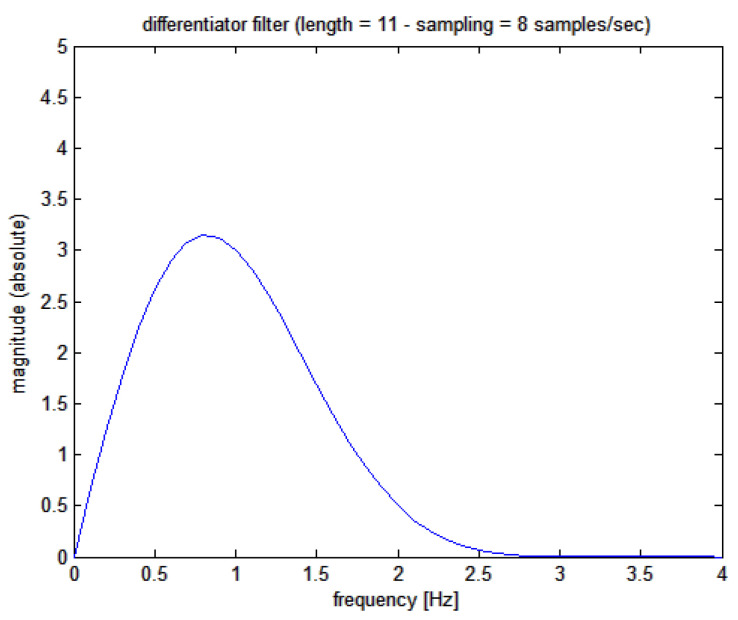
Magnitude Bode plot of Holoborodko’s differentiating filter applied to the cumulative sum signal. Please note the differentiating behavior at low frequencies thanks to the almost straight line of the graph (the output is linearly proportional to the frequency of the input signal).

**Figure 6:**
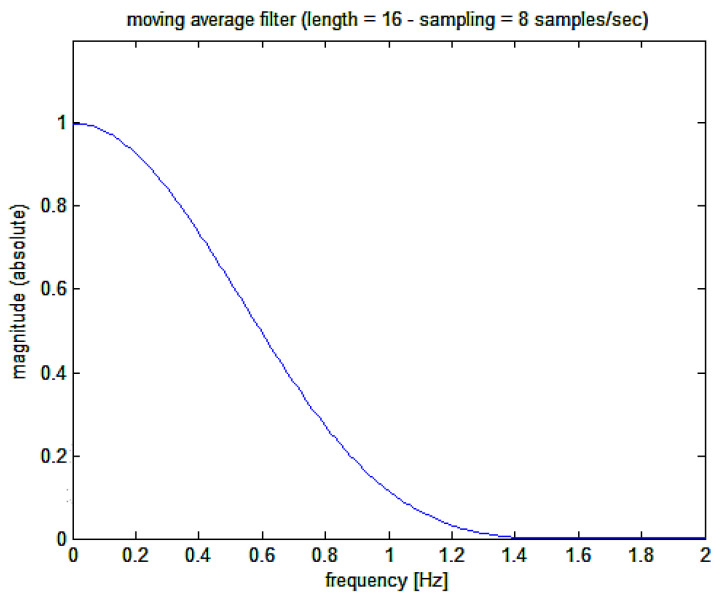
Magnitude Bode plot of the moving average filter applied to the recovered first derivative signal (the final recovered heart rate signal).

The obtained signal is not directly helpful in estimating its first derivative, because it is composed of a series of steps (of a staircase shape) of one count for each cycle, or heartbeat, found. The numerical first derivative is quite a tricky procedure in terms of obtaining a sufficiently smooth and low-noise output signal, and therefore the following steps are taken:

(a) Initially, the SR(t) signal is low-pass filtered with a moving average 256 points on a Kaiser-Bessel weighted filter, whose Bode plot is shown in Figure 4 (corresponding, at the sampling frequency used of 128 Hz, to a 2 s epoch and giving a filter delay of 1 s (To recall a basic signal theory concept: the time delay $t_{d}$ for a FIR filter having N taps (points or coefficients) at a sampling frequency $f_{s}$ is equal to $t_{d}=\left(N-1\right)/\left(2\cdot f_{s}\right)$.))

(b) Next, a downsampling of the SR(t) signal is made to a sampling frequency of 8 Hz, as the maximum bandwidth of the HRV signal is expected to be approximately 0.5 Hz at most (30 cycles per minute)

(c) Eventually, filtering is applied to the downsampled SR(t) signal with a differentiating noise-robust filter to obtain the first derivative HR(t) or the instantaneous heart rate signal; signal differentiation by FIR filtering is a classical procedure, and therefore the original and robust method proposed by Holoborodko [23] was followed using an 11-order one-sided noise-robust differentiator whose Bode plot is shown in Figure 5; a filter delay of 625 ms is expected. This filter has a linear characteristic at low frequencies (implying a multiplication by the s-variable in the Laplace domain), while at higher frequencies it becomes a low pass.

(d) A final low pass filter is then applied to the HR(t) signal with a moving average Kaiser-Bessel 16-point weighted filter whose Bode plot is shown in Figure 6 (corresponding, at the sampling frequency of 8 Hz, to approximately 2 s and giving a filter delay of 937.5 ms); following the differentiating filter, which provides the required first derivative. This moving average filter is then used to clean the output from residuals of the original R-wave detection points (the original carrier frequency).

(e) Eventually, the average value of HR(t) is subtracted from itself to obtain the final HRV(t) signal or the heart rate variation signal.

**Figure 7:**
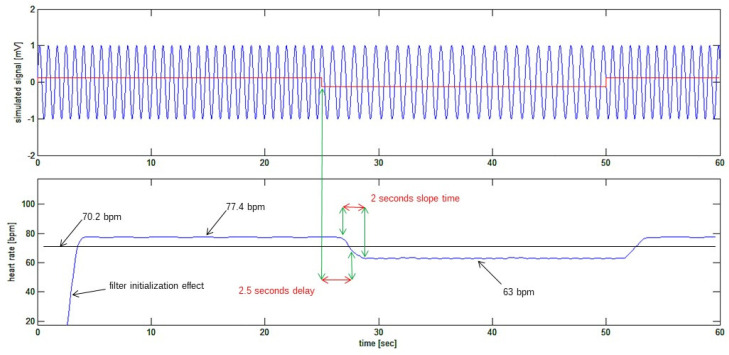
Bottom trace: the recovered heart rate signal. The 2.5-s delay of the recovered signal corresponds to the delays expected from the sequence of FIR filters.

Finally, the heart rate signal can be plotted, as is shown in Figure 7. This figure shows how the recovered heart rate signal (bottom trace) closely resembles the simulated signal (top trace in red), which was used to modulate the frequency of the “sinusoidal ECG”. In particular, the step from 77.4 bpm to 63 bpm has been detected with a 2.5-s delay (known from the fixed delays applied by the filters), and the sudden step in the heart rate is detected with a 2-s gentle slope of the recovered signal, which is about two heart cycles in time length. The beginning of the reconstructed signal is affected by the initialization transition period of reconstructing filters or edge effects.

## 3. Results I: Testing the Algorithm on More Complex Artificially Generated Heart Rate Variability Signals

Software implementation of the simulation in Figure 2 is relatively straightforward, as the frequency-varying sinusoidal signal is composed of epochs in which its frequency is constant (in the example, there are epochs in which the frequency is 77.4 bpm, alternating with epochs at 63 bpm). However, this situation is not what one might expect in general from the frequency wandering of the natural ECG; indeed, in nature, what is expected is a continuous variation of the instantaneous frequency of the ECG. We then put it into formulas. The sinusoidal signal to be used must show continuous frequency wandering behavior, as can be seen in an actual ECG. A sinusoidal signal instead of an ECG-shaped signal will be used. From secondary school, the sinus function is presented as: sin(ω⋅t)=sin(2⋅π⋅f⋅t), which is strictly an accurate periodical signal of period 2⋅π. For the scope of introducing frequency wandering in the formula, one must re-learn what a sinus function is all about: Given a time domain function HR(t) representing the instantaneous value of the frequency of the sinus signal, or heart rate, this can be represented in a formula, as follows:

(3) sin(2⋅π⋅(∫HR(t)⋅dt))

Should HR(t) be reduced to a constant f not depending on time, then the formula above will become the well-known formula for the actual periodic (not frequency wandering) sinus function:

(4) sin(2⋅π⋅(∫HR(t)⋅dt))=sin(2⋅π⋅(∫f⋅dt))=sin(2⋅π⋅f⋅t)

Of course, as the frequency of our “heart sinusoidal signal” is not constant, the first representation as in Formula (3) above is kept.

Following the example in Figure 2, the function HR(t) is a time-domain signal whose amplitude is the instantaneous heart rate or the instantaneous frequency of the ECG signal. As a matter of fact, the signal HR(t) is considered to be composed of two parts, one constant and another variable in time, such as:

(5) $$HR\left(t\right)=\bar{f_{0}}+HRV\left(t\right)$$

where $\bar{f_{0}}$ is the constant average value of HR(t), and the function HRV(t) is the variable component or, more appropriately, the heart rate variability signal that was sought. In the example simulated in Figure 2, the following values are used:

$\bar{f_{0}}=70$ bpm, and $HRV\left(t\right)=7.2\cdot sign\left(sin\left(2\cdot \pi \cdot f_{t}\cdot t\right)\right)$ bpm

where sign(t) is the sign function, and $f_{t}$ is the frequency of variation of the instantaneous signal frequency (0.02 Hz, meaning the heart rate is changing every 25 s).

In a more general case, $\bar{f_{0}}$ will be defined as the average (constant) heart rate during a given (short) epoch of signal acquisition, and HRV(t) will be defined as the wandering frequency signal, or the heart rate variability signal, whose amplitude is the instantaneous shift of the heart rate from the average heart rate (frequency modulation).

The problem of recording the HRV(t) signal can be stated as the method by which this signal can be inferred from the quasi-periodic signal ECG(t). The need to obtain a “continuous”, or “instantaneous”, HR(t) signal comes from the interest in analyzing very short or even ultra-short-term heart rate variations in the time domain, instead of considering cyclical variations over very long periods of time.

To describe the performance of the method described in the previous paragraph, simulated signals were initially used for which the signal used for simulating them was exactly known. It must be noted that the use of simulated signals for testing the algorithm is necessary because in this way one can compare the results of the method with the signal actually used in the simulations. For the sake of validation, the method could not be applied to real ECG signals because, in this case, the real frequency variability coded into the heart rate was not known. Once the method is validated on simulated signals, it will then be reliably used on real-life natural signals. Eventually, the method will be applied to real ECG biological signals, as described in Section 4.

For the HRV(t) signal, a square waveform was used, as was already shown in Figure 2, and now the performance and results of the algorithm on a mix of superimposed sinusoidal signals of different frequencies will be described.

Initially, one restates:

(6) $$HR\left(t\right)=\bar{f_{0}}+HRV\left(t\right)$$

then, from (3):

$$ECG\left(t\right)=sin\left(2\cdot \pi \cdot \left(\int \left(\bar{f_{0}}+HRV\left(t\right)\right)\cdot dt\right)\right)$$

(7) $$ECG\left(t\right)=sin\left(2\cdot \pi \cdot \bar{f_{0}}\cdot t+2\cdot \pi \cdot \left(\int HRV\left(t\right)\cdot dt\right)\right)$$

In this simulation it will be considered that:

$\bar{f_{0}}$ (Hz) or $60\cdot \bar{f_{0}}$ (bpm) as the average heart rate, and

(8) $$HRV\left(t\right)=f_{s1}\cdot sin\left(2\cdot \pi \cdot f_{HRV1}\cdot t\right)+f_{s2}\cdot sin\left(2\cdot \pi \cdot f_{HRV2}\cdot t\right)$$

as a heart rate variability signal produced by the sum of two sinusoidal waves with two different amplitudes and two different frequencies. The following values are assumed for the various parameters:

$\bar{f_{0}}=1.17$ Hz or 70.2 bpm, $f_{s1}=0.06$ and $f_{s2}=0.12$ as the modulation width of the two modulating signals, and $f_{HRV1}=0.19$ Hz and $f_{HRV2}=0.32$ Hz as the modulation frequencies of the two modulating signals.

The indicated parameters of the simulated ECG signal will have the following expression:

(9) $$ECG\left(t\right)=sin\left(2\cdot \pi \cdot \left(\int \left(\bar{f_{0}}+f_{s1}\cdot sin\left(2\cdot \pi \cdot f_{HRV1}\cdot t\right)+f_{s2}\cdot sin\left(2\cdot \pi \cdot f_{HRV2}\cdot t\right)\right)\cdot dt\right)\right)$$

(10) ECG(t)=sin(2⋅π⋅(∫(1.17+0.06⋅sin(2⋅π⋅0.19⋅t)+0.12⋅sin(2⋅π⋅0.32⋅t))⋅dt))

and, solving the integral:

(11) $$ECG\left(t\right)=sin\left(2\cdot \pi \cdot 1.17\cdot t-\frac{0.06}{2\cdot \pi \cdot 0.19}cos\left(2\cdot \pi \cdot 0.19\cdot t\right)-\frac{0.12}{2\cdot \pi \cdot 0.32}cos\left(2\cdot \pi \cdot 0.32\cdot t\right)\right)$$

finally:

(12) ECG(t)=sin(7.3513⋅t−0.0502⋅cos(1.1938⋅t)−0.0597⋅cos(2.0106⋅t))

**Figure 8:**
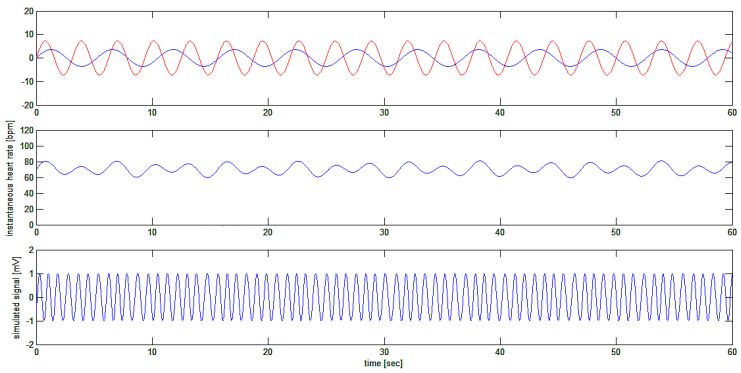
Simulation of test signal ECG(t): a sinusoidal signal (bottom trace) that is frequency modulated with the sum of two sinusoidal waves (shown separately in different colors on the upper trace), producing a final modulating signal around the average frequency of 70.2 bpm (middle trace).

Thus, the signal ECG(t) is a sinusoidal signal with a sinusoidal periodic variation of its frequency given by the sum of the two sinusoidal frequency modulating signals, as shown in Figure 8.

The sinusoidal carrier with an average frequency of 70.2 bpm is modulated by two sinusoidal signals at 0.19 Hz (11.4 cycles per minute) and 0.32 Hz (19.2 cycles per minute), respectively, with modulation depths of 3.6 and 7.2 cycles per minute, respectively.

**Figure 9:**
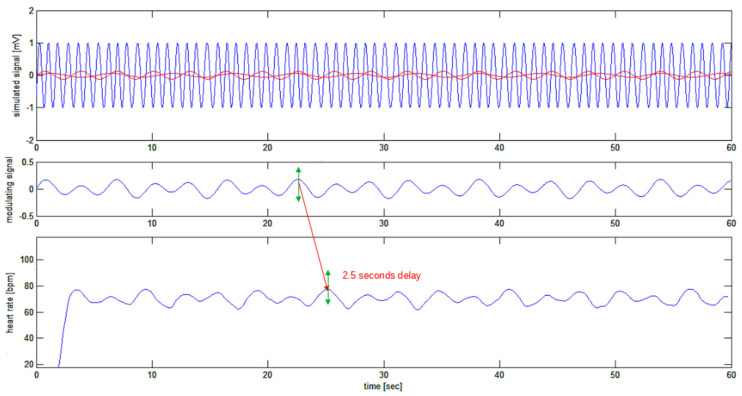
Upper trace: frequency-modulated signal and modulating signal. Bottom trace: the recovered heart rate signal. The middle trace represents the modulating waveform (cf. Figure 8).

The application of the algorithm on this complex simulated signal, which better represents the actual situation found in nature, provides the results depicted in Figure 9.

**Figure 10:**
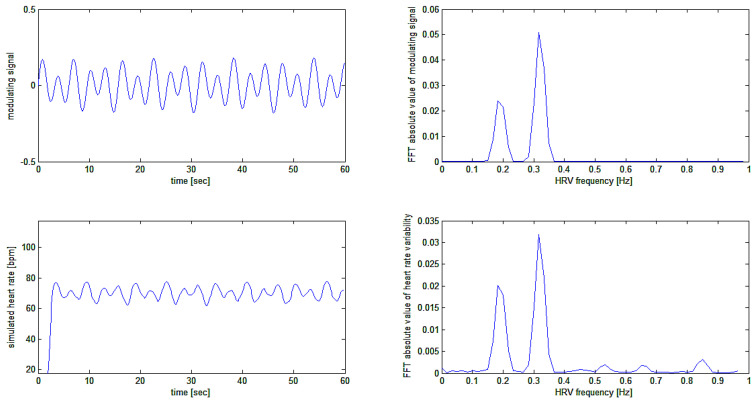
A comparison of modulating and recovered simulation signals in the time and frequency domains. The HRV frequencies are perfectly recovered. Different amplitudes of signals in the time domain result from changing the scale from beats per second to beats per minute. Different amplitudes in the frequency domain are also due to the windowing effect (a Blackman-Harris window was used before the FFT calculation).

In an attempt to verify the quality of modulation recovery (i.e., the heart rate variability signal) from the simulated heart rate signal, the Fast Fourier Transform (FFT) of the original modulating signal and of the recovered modulation signal is calculated. The FFT was computed on the simulation, as shown in Figure 9 above, and thus on the middle and bottom tracings of Figure 9 above. The results are plotted in Figure 10.

## 4. Results II: Analysis of Real ECG Signals Epochs for the Appreciation of Ultra-Short-Term HRV

As the purpose of the present work was not to analyze ECG signals for HRV estimation, but rather to explain a method for doing so, the algorithm was initially tested on a few epochs of the ECG of one of the authors. No human subject was used for acquiring ECG tracings, other than the authors themselves; this is merely to show the application of the method on real tracings.

**Figure 11:**
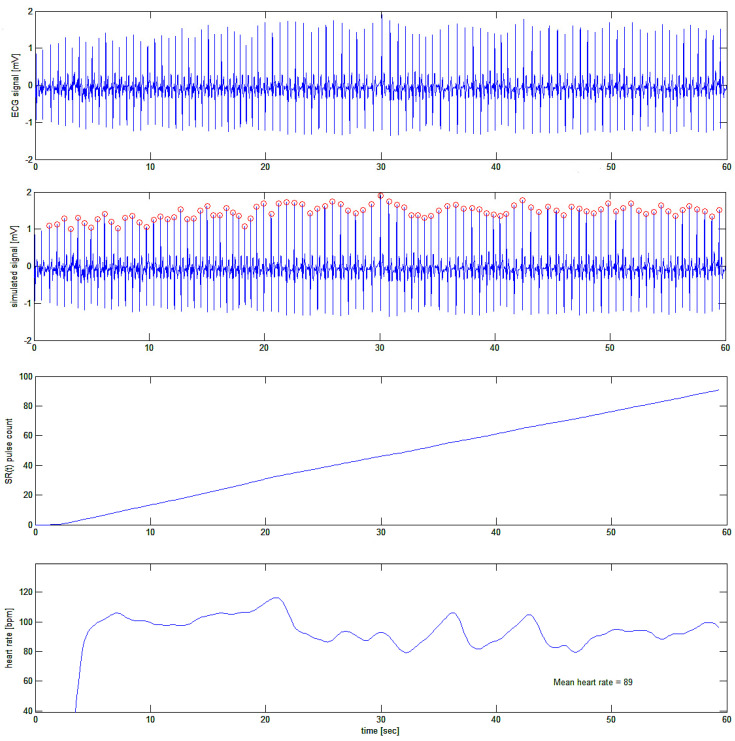
First test of the algorithm on a real ECG trace (top trace). In the second tracing from the top, ECG R-waves are indicated as being detected. In the third tracing, the cumulative summing signal is shown, and in the bottom trace, the filtered first derivative of the latter is plotted showing fast variations of the heart rate during the subject’s activity.

In Figure 11, the ECG of one of the authors (H.K.) was taken while he was busy playing a computer game. The idea was to acquire the fast variations in heart rate (if any) during the cognitive and emotional effort of computer gaming. Again, the authors are only interested in the ECG tracing per se, and at this stage of the research, no interest was given to the meaning of HRV signals or their correlation with the activity of the subject. Further papers will address this by using the present algorithm.

There are many different, robust, and efficient algorithms for the real-time detection of the R-wave [24,25,26,27]. In this work, a very simple software detection based on adaptive amplitude and time thresholds was programmed. The resulting peak finder and R-wave detector function were written in MATLAB as follows:
function pf = RWaveFind(y,yth,tth) % % RWAVEFIND Find all the peaks in the values passed on y %   RWAVEFIND (y, yth, tth) %   where each valid peak found must have a value above yth and %   must be more distant than tth indexes from last found peak %   returns a two columns matrix %    first column are indexes where peaks were found %    second column are amplitudes of peaks found % pf=[];  % start with an empty output matrix iold=1;  % sampling index where last peak found for i=2:(size(y,2)-1) % explore all samples on the input vector   if (y(i-1)<y(i)) && (y(i+1)<y(i)) && (y(i)>yth)        % test if current sample is a peak   td=i-iold; % calculate time delay from last peak found   iftd>tth % test if new time delay too short     pf=[pf; i y(i)]; % add time delay to output vector     iold=i; % keep memory of new peak found     yth=0.5*y(i); % threshold adapting to half last   end  end end

As the underlying scope of our research is that of implementing a method for assessing vagus nerve activity using the heart (heart rate) as a sort of “vagus sensor” with the final ambitious goal of recording “vagus nerve event-related activity”, the method was preliminary used on one of the well-known vagal maneuvers like the Valsalva maneuver. The fact that this research did not involve any clinical research on humans is worthy of note; the newly developed algorithm was only used on the author’s own ECG tracings (the one of the authors) to look at a real HRV signal out of the simulations, which permitted the testing of the method “in vitro”, as explained previously.

The various methods used to stimulate the vagus nerve are well-known and have been assessed clinically. They all go by the name of vagal maneuvers. The most clinically useful are the Valsalva maneuver, the carotid sinus massage, the cold-water immersion, and the eyeball pressure. The effects of these maneuvers are different, but they all provoke an increase or decrease in the heart rate, which is often mediated by a concurrent vagal stimulation.

The Valsalva maneuver was chosen as the simplest way to qualitatively test our method in the lab. It was performed by making a forced expiratory effort against a closed airway, and is considered to be a very low-risk procedure [28,29,30].

**Figure 12:**
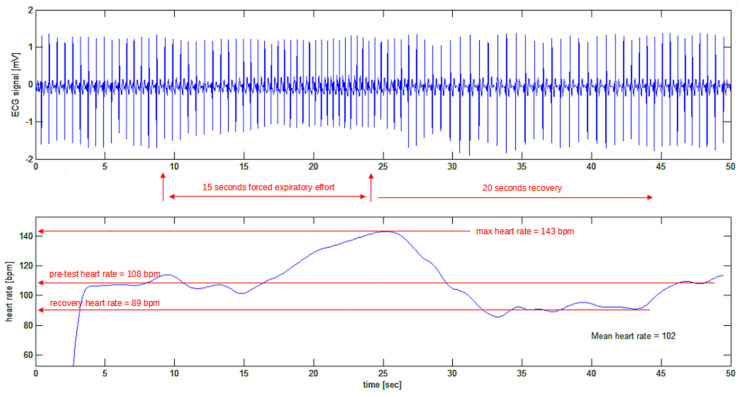
Test of the algorithm on a real ECG trace (top trace) during the Valsalva manoeuvre.

The ECG tracings were recorded using a custom-made two-channel ECG [31]. The second channel was used for recording marks for the different phases of the Valsalva maneuver, namely the exact beginning and ending times of the forceful attempt at exhalation. The ECG tracing that was obtained was processed using the algorithm, and the results are shown in Figure 12.

In Figure 12, a typical tracing is shown where the resting heart rate of the subject (one of the authors: H.K.) is 108 bpm. At the end of the forced expiratory effort, the heart rate went up to 143 bpm (or 35 bpm above the resting value). During the recovery phase, the heart rate went down to 89 bpm in just 7 s (or 54 bpm down with a negative slew rate of 8 bpm per second) before restoring the resting heart rate to 108 bpm.

## 5. Discussion

Most readers with a working experience in real-time heart rate detection for HRV studies or other research purposes, know very well how an instantaneous heart rate can be inferred from the common interbeat graph plotting the heart period or the inverse of it, in addition to the heart rate at each detected beat (or R-wave).

As already highlighted, the resulting instantaneous heart rate signal is not evenly sampled, and thus the interpolation, the smoothing, or both need to be used to obtain an evenly sampled heart rate. 

In the previous paragraph, the importance of the new method is stressed to show how the proposed method is by itself able to detect the heart rate without resolving to measure the instantaneous beat by beat heart period, while at the same time maintaining an evenly sampled heart rate signal.

Nevertheless, as a comparison, we used the common interbeat analysis to show the different performances and to compare the results of the method presented in this paper with the more common one.

**Figure 13:**
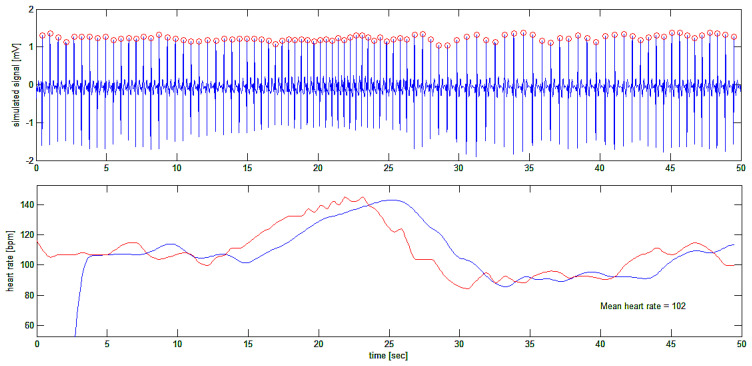
Test of the algorithm on a real ECG trace, as was shown in Figure 12. Bottom blue trace: heart rate signal obtained with the presented algorithm; bottom red trace: heart rate signal obtained with common interbeat calculation followed by even sampling with cubic interpolation. The time delay of the blue trace is due to the numerical filtering delay and is explained in the text.

In Figure 13, the heart rate signal obtained with the presented algorithm (blue trace) is compared with the more common heart rate signal obtained with interbeat calculation (red trace), and was evenly sampled using interpolation (cubic interpolation was used, as it provides the best results). While the two reconstructed heart rate curves are similar, it must be noted that the interbeat curve showed fast oscillatory artifacts due to the interpolation of subsequent slightly different periods of the heart rate.

In addition, the reconstructed heart rate using the present algorithm preserves the shape of the heart rate variation in time, while avoiding artifacts. It should be pointed out that, because of this, the common interbeat curve is only used on very long periods of time on the ECG, as a much harder interpolation and low-pass filtering must be done to obtain an acceptable heart rate signal. In this way, the processing is only useful for analysing the heart rate on epochs of minutes, thus losing the rapid variations of the heart rate itself.

The merits of the new method lie in the possibility to reliably follow the rapid variations of the heart rate which go unnoticed with more common and still largely used methods. The relatively new interest in ultra-short-time heart rate variability [32,33] may benefit from the use of the heart rate signal detection presented here.

## 6. Conclusions

The proposed method for HRV signal detection has already been well-described and tested extensively on simulated signals and real ECG tracings, which produced consistent results. Discussion text has already been inserted into the various paragraphs dealing with the description of the algorithm and its applications. In this regard, particular attention should be given to the comparison of our method with other, more conventional methods of HRV detection. The superiority of this method has been demonstrated, as it completely avoids any suspicious activity of resampling or interpolation on the RR periodogram or interbeat interval tracing. In addition, a form of filtered interpolation remains to be detected in the use of low pass filtering before and after the differentiation of the cumulative sum signal.

Nevertheless, the method appears to provide a very clean HRV signal which maintains its frequency content, even at high frequencies.

The method might also be used on other frequency-modulated biomedical signals to extract a reliable modulation signal embedded in them.

## Data Availability

Data used for the simulations are available from the corresponding author.
